# Seroprevalence of Severe Fever with Thrombocytopenia Syndrome Virus Antibodies in Rural Areas, South Korea

**DOI:** 10.3201/eid2405.152104

**Published:** 2018-05

**Authors:** Mi Ah Han, Choon-Mee Kim, Dong-Min Kim, Na Ra Yun, Sun-Whan Park, Myung Guk Han, Won-Ja Lee

**Affiliations:** Chosun University College of Medicine, Chosun University, Gwangju, South Korea (M.A. Han, C.-M. Kim, D.-M. Kim, N.R. Yun);; Korea National Institute of Health, Cheongju, South Korea (S.-W. Park, M.G. Han, W.-L. Lee)

**Keywords:** seroprevalence, antibodies, severe fever with thrombocytopenia syndrome, SFTS, severe fever with thrombocytopenia syndrome virus, SFTSV, viruses, ticks, vector-borne infections, rural areas, South Korea

## Abstract

We investigated 1,228 residents of 3 rural areas in South Korea and determined that 50 (4.1%) were positive for severe fever with thrombocytopenia syndrome virus antibodies. Fever and gastrointestinal symptoms in the previous 3 years and career duration were associated with virus seropositivity.

Severe fever with thrombocytopenia syndrome (SFTS) is a tick-transmitted, acute febrile disease caused by SFTS virus (SFTSV) (*1**,**2*). Previous studies have not determined the seroprevalence of SFTSV in South Korea. Therefore, we investigated SFTSV seroprevalence among residents of rural areas in South Korea and identified factors associated with seropositivity.

## The Study

**Figure 1:**
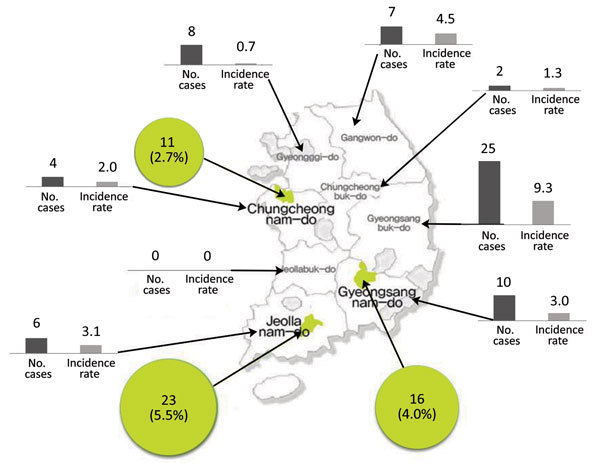
Seroprevalence in 3 rural areas (2014) and incidence in 8 provinces (2013–2014) of severe fever with thrombocytopenia syndrome, South Korea. Within each province, 1 rural area was selected on the basis of the number of cases. Green circles indicate seroprevalence determined by using an indirect immunofluorescence assay. The incidence rate is per 1 million persons.

**Figure 2:**
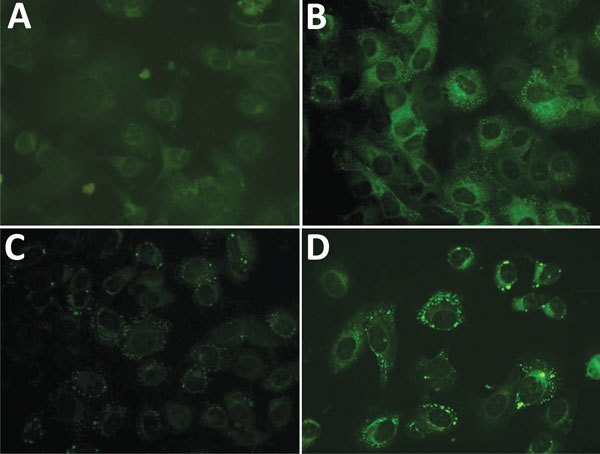
Representative indirect immunofluorescent assays of Vero E6 cells infected with thrombocytopenia syndrome virus from patients in rural areas, South Korea. Indirect immunofluorescent assays were conducted by using serially diluted patient serum as primary antibody and fluorescein isothiocyanate–conjugated antihuman IgG as secondary antibody. A) H1 serum (negative, dilution 1:32, IgG titer <1:32); B) B321 serum (positive, dilution 1:64, IgG titer 1:512); C) H214 serum (positive, dilution 1:32, IgG titer 1:128); D) D127 serum (positive, dilution 1:32, IgG titer 1:256). Original magnification x400.

We conducted our study in rural areas of 3 provinces (Myeoncheon-myeon, Dangjin-gun, Choongcheongnam-do Province; Nodong-myeon, Boseong-gun, Jeollanam-do Province; and Gahoe-myeon, Hapcheon-gun, Gyeosangnam-do Province) in South Korea that had reported SFTS patients to the Korean Center for Disease Control during 2013–2014 (Figure 1) (*3*). In September 2014, we administered a structured questionnaire regarding demographic characteristics and occupational and living conditions to 1,228 residents of these areas. We collected blood samples from these persons and subjected them to indirect immunofluorescent assays to determine SFTSV IgG titers. Participants were defined as being seropositive if the indirect immunofluorescent assay IgG titer was >1:32 (Figure 2). Written consent was obtained from all participants before administration of the survey and blood tests. This study was approved by the Chosun University Institutional Review Board.

Of 1,228 persons included in the analysis (Table 1), 786 (64.0%) were women, 831 (67.7%) were >65 years of age, and 713 (58.1%) worked in agriculture. A total of 225 (18.3%) participants had lived in the same residence for 1–20 years, and 757 (61.6%) had lived in the same residence for >41 years; 255 (20.8%) had raised domestic animals and livestock (among which dogs were most common). Furthermore, 166 (13.5%) had received a tick bite during their lifetime (Table 1). The highest tick bite rate was reported in Boseong (94, 26.8%), followed by Hapcheon (43, 10.7%) and Dangjin (29, 7.1%). In the year before the study, 75 (6.1%) of patients in the total cohort had a tick bite (Boseong: 52, 12.5%; Dangjin: 13, 3.2%; Hapcheon: 10, 2.5%), and 25 (2.0%) had a fever and gastrointestinal (GI) symptoms (i.e., SFTS symptoms) during the previous 3 years.

**Table 1:** Epidemiologic characteristics for 1,228 patients with suspected SFTS in rural areas, South Korea*

Characteristic	Total, no. (%)	Seronegative, no. (%)	Seropositive, no. (%)	p value†
No. patients	1,228 (100.0)	1,178 (95.9)	50 (4.1)	
Area				0.116
Bosung	416 (33.9)	393 (94.5)	23 (5.5)	
Dangjin	411 (33.5)	400 (97.3)	11 (2.7)	
Hapcheon	401 (32.7)	385 (96.0)	16 (4.0)	
Sex				
M	442 (36.0)	425 (96.2)	17 (3.9)	0.764
F	786 (64.0)	753 (95.8)	33 (4.2)	
Age, y				0.027
<64	397 (32.3)	388 (97.7)	9 (2.3)	
>65	831 (67.7)	790 (95.1)	41 (4.9)	
Education level				0.489
Uneducated (illiterate)	318 (25.9)	303 (95.3)	15 (4.7)	
Uneducated (literate)	165 (13.5)	158 (95.8)	7 (4.2)	
Elementary school	361 (29.5)	343 (95.0)	18 (5.0)	
Middle school	193 (15.7)	187 (96.9)	6 (3.1)	
More than high school	189 (15.4)	185 (97.9)	4 (2.1)	
Smoking status				0.324
Never	876 (71.4)	845 (96.5)	31 (3.5)	
Former	205 (16.7)	194 (94.6)	11 (5.4)	
Current	146 (11.9)	138 (94.5)	8 (5.5)	
Drinking frequency, no. drinks				0.587
None	712 (58.0)	683 (95.9)	29 (4.1)	
<1/mo	164 (13.4)	159 (97.0)	5 (3.1)	
<3/wk	100 (8.1)	93 (93.0)	7 (7.0)	
4–6/wk	148 (12.1)	143 (96.6)	5 (3.4)	
Every day	104 (8.5)	100 (96.2)	4 (3.9)	
Chronic disease				0.492
None	318 (25.9)	306 (96.2)	12 (3.8)	
1	417 (34.0)	403 (96.6)	14 (3.4)	
>2	493 (40.2)	469 (95.1)	24 (4.9)	
Residency duration, y				0.023
1–20	225 (18.3)	221 (98.2)	4 (1.8)	
21–40	246 (20.0)	240 (97.6)	6 (2.4)	
>41	757 (61.6)	717 (94.7)	40 (5.3)	
No. family members				0.074
0 (alone) or 1	954 (77.7)	910 (95.4)	44 (4.6)	
>2	274 (22.3)	268 (97.8)	6 (2.2)	
Raising livestock‡				0.352
No	973 (79.2)	936 (96.2)	37 (3.8)	
Yes	255 (20.8)	242 (94.9)	13 (5.1)	
Grazing livestock				0.165
Yes	119 (9.7)	117 (98.3)	2 (1.7)	
No	1109 (90.3)	1061 (95.7)	48 (4.3)	
Occupation				0.437
Agriculture	713 (58.1)	680 (95.4)	33 (4.6)	
Stock farming	31 (2.5)	30 (96.8)	1 (3.2)	
Housewife	85 (6.9)	80 (94.1)	5 (5.9)	
Farm housewife	160 (13.0)	154 (96.3)	6 (3.8)	
Other	239 (19.5)	234 (97.9)	5 (2.1)	
Career duration, y				0.006
1–20	433 (35.3)	423 (97.7)	10 (2.3)	
21–40	256 (20.9)	249 (97.3)	7 (2.7)	
>41	539 (43.9)	506 (93.9)	33 (6.1)	
Tick bite during lifetime				0.171
Yes	166 (13.5)	156 (94.0)	10 (6.0)	
No	1,062 (86.5)	1,022 (96.2)	40 (3.8)	
Tick bite during previous year				0.076
Yes	75 (6.1)	69 (92.0)	6 (8.0)	
No	1,153 (93.9)	1,109 (96.2)	44 (3.8)	
Awareness of SFTS				0.446
Completely unaware	500 (40.8)	477 (95.4)	23 (4.6)	
Somewhat aware	558 (45.5)	535 (95.9)	23 (4.1)	
Completely aware	169 (13.8)	165 (97.6)	4 (2.4)	
History of SFTS or febrile illness during the fall				0.575
Yes	97 (7.9)	92 (94.9)	5 (5.2)	
No	1,130 (92.1)	1,085 (96.0)	45 (4.0)	
Family history of SFTS or febrile illness during the fall				0.235
Yes	56 (4.6)	52 (92.9)	4 (7.1)	
No	1,171 (95.4)	1,125 (96.1)	46 (3.9)	
SFTS-related symptoms during the previous 3 y				0.002
Yes	25 (2.0)	21 (84.0)	4 (16.0)	
No	1,202 (98.0)	1,156 (96.2)	46 (3.8)	

*SFTS, severe fever with thrombocytopenia syndrome. †By χ^2^ test. ‡Including cattle, goats, sheep, deer, and birds.

Among the total sample, 50 (4.1%) persons were seropositive for SFTSV (Figure 1): 23 (5.5%) in Boseong, 16 (4.0%) in Hapcheon, and 11 (2.7%) in Dangjin. Antibody seroprevalence was 2.3% (9/397) for persons <64 years of age and 4.9% (41/831) for persons >65 years of age (Table 1). Persons who had fever and GI symptoms in the previous 3 years were more likely to be seropositive. Antibody positivity was also higher for persons with a long career duration, but we did not identify any correlations with occupation type, outdoor activity–related characteristics, or type of work in the previous year. Furthermore, 50 persons had antibody titers >1:32, among whom 6 had had a tick bite in the previous year. Among these 6 persons, 5 (83.3%) had an antibody titer >1:128. For 44 persons who did not have tick bites in the previous year, 15 (34.1%) had an antibody titer >1:128. Persons who had a tick bite in the past year had significantly higher antibody titers (p = 0.021).

We used multiple logistic regression analysis to identify variables with significant (p<0.1) probabilities of being associated with seropositivity. Persons who had fever and GI symptoms in the previous 3 years (odds ratio [OR] 4.09, 95% CI 1.25–13.36) and those who had a career duration of >41 years (OR 2.36, 95% CI 1.11–5.02) had a higher likelihood of seropositivity than nonsymptomatic persons and those who had a career duration of 1–20 years (Table 2). In addition, of 25 (2%) persons with fever and GI symptoms or suspected SFTS symptoms in the previous 3 years, 4 (16.0%) were SFTS seropositive; among the 1,202 with no suspected SFTS symptoms, 3.8% (n = 46) were SFTS seropositive (p = 0.002). Persons who had fever and GI symptoms in the previous 3 years were more likely to be seropositive for SFTSV.

**Table 2:** Risk factors associated with SFTSV seropositivity determined by using multiple logistic regression in rural areas, South Korea*

Characteristic	aOR (95% CI)
Age, y	
<64	1.00
>65	1.45 (0.60–3.50)
No. family members	
0 (alone) or 1	1.57 (0.64–3.87)
>2	1.00
Tick bite during previous year	
No	1.00
Yes	1.60 (0.62–4.11)
SFTS-related symptoms during previous 3 y	
No	1.00
Yes	4.09 (1.25–13.36)
Career duration	
1–20	1.00
21–40	1.44 (0.52–3.99)
>41	2.36 (1.11–5.02)

*aOR, adjusted odd ratio; SFTSV, severe fever with thrombocytopenia syndrome virus.

## Conclusions

A much higher mortality rate was observed for patients with SFTS in South Korea than for patients in China (*4*). In addition, the high SFTS case-fatality rate in South Korea (47.2%, 17/36) is a serious public health concern (*5*). The 3 areas examined in this study were rural, and most residents were elderly agriculture workers. In China, queried seropositive farmers denied having typical SFTS symptoms (*6*). In contrast, despite possible recall bias in our study, seroprevalence was higher for patients who reported fever and GI symptoms (i.e., SFTS symptoms) during the previous 3 years.

When 2,510 residents of Jiangsu Province, China, were subjected to SFTSV antibody testing, 1,104 (0.44%) were seropositive (*6*). In contrast, a study of 2,547 farmers in a rural area of the same province reported a seropositivity rate of 1.3% (*7*). Thus, SFTSV seroprevalence was 3-fold higher for rural farmers than for the general population (*6**,**7*). Li et al. also reported that seroprevalence tended to increase with age (*7*). These findings might help to explain the relatively high seroprevalence (4.1%) observed in our study.

Moreover, studies have indicated that mild or subclinical SFTS might be common. For example, SFTS viral antibody testing of healthy residents in Zhejiang Province, China, showed that 7.2% had IgG against SFTSV (*8*). In this study, when persons were asked if they had had GI symptoms and fever in the previous 3 years, which indicated a suspected SFTSV infection, 25 (2.0%) persons, including 4 (8.0%) of 50 seropositive persons reported that they had had these suspected SFTS symptoms. Furthermore, persons who reported suspected SFTS symptoms were more likely to be seropositive. Therefore, subclinical or mild SFTSV infections might be present in the study communities.

In addition, the higher antibody titers for persons who had ticks bites in the past year indicate a correlation between tick bites and antibody titer positivity. Long career duration was associated with SFTS seroprevalence in this study. Most study participants were elderly; age and career duration showed a weak positive correlation (r = 0.312, p<0.001). Additional research is needed to investigate SFTSV seroprevalence, including various age groups containing an adequate number of persons. 

Our study had some limitations. First, we did not use other seropositivity testing methods, such as ELISA, because of lack of availability. Second, recollection of symptoms (e.g., fever) on the questionnaire might have introduced recall bias.

In summary, SFTSV seroprevalence was 4.1% for residents in 3 rural areas of South Korea. A history of fever and GI symptoms and a long career duration were associated with SFTSV seroprevalence.
